# Inferring resource use from functional area presence in a small, single-flock of chickens in a mobile barn

**DOI:** 10.1016/j.psj.2024.104123

**Published:** 2024-07-27

**Authors:** Serge Alindekon, Jana Deutsch, Jan Langbein, T. Bas Rodenburg, Birger Puppe, Timo Homeier-Bachmann, Helen Louton

**Affiliations:** ⁎ Animal Health and Animal Welfare, Faculty of Agricultural and Environmental Sciences, University of Rostock, 18059 Rostock, Germany; † Behaviour and Welfare, Research Institute for Farm Animal Biology (FBN), 18196 Dummerstorf, Germany; ‡ Animals in Science and Society, Faculty of Veterinary Medicine, Utrecht University, 3584 CM Utrecht, The Netherlands; § Behavioral Sciences, Faculty of Agricultural and Environmental Sciences, University of Rostock, 18059 Rostock, Germany; ║ Institute of Epidemiology, Friedrich-Loeffler-Institut (FLI), Federal Research Institute for Animal Health, 17493 Greifswald - Insel Riems, Germany

**Keywords:** resource use, poultry behavior, tracking technology, precision livestock farming

## Abstract

In poultry behavior research, the reliance on presence data to estimate actual resource usage has substantially increased with the advent of tracking technologies such as radio frequency identification (**RFID**) and image-based systems. Although such widely used technologies are fundamentally designed for presence tracking, many studies claim to use them to investigate actual resource usage. This study investigates whether the duration of chickens' presence near key resources accurately reflects their actual usage. To this end, we analyzed 210 ten-min video sequences from 5 days of recordings of 21 chickens, focusing on their proximity to and use of 6 key resources in a mobile poultry barn. Human observers manually assessed the durations of proximity—presence in defined functional areas of interest—and resource use for each individual in the video sequences. Significant correlations (Spearman's coefficient 0.83–1) were found for most resources, except the pophole (Rho = −0.30). Usage-to-presence ratios varied: perches exceeded 87%, feeder and enrichments around 66%, drinker 50%, and pophole 10%. Our findings highlight that mere proximity to resources does not always guarantee their effective use. We emphasize the need for careful interpretation of data from tracking technologies, acknowledging the distinction between mere proximity and actual resource use. Future studies should include larger sample sizes and varied conditions to ensure broader applicability.

## INTRODUCTION

Understanding chicken behavior, particularly their interactions with key resources such as drinkers, feeders, and perches, helps to develop housing and management practices that meet their needs, reduce stress, and promote positive human-animal relationships, ultimately optimizing costs and farm profitability.

Recent advancements in tracking technologies have significantly enhanced the study of chicken behavior, providing valuable spatiotemporal data on resource use and spatial distribution (Neves et al., 2015; Alindekon et al., 2023). However, technologies like RFID and basic image-based tracking, which are fundamentally designed for presence tracking, may often fail to differentiate mere proximity from actual effective resource use (Doornweerd et al., 2023). When used alone, RFID detects an animal's presence by sensing changes in electromagnetic fields, while image-based systems, relying on pixel changes, can only indicate presence within an area of interest (**AOI**). Neither method can accurately depict specific animal actions without additional technologies or measures. Despite this limitation, many studies (reviewed by Alindekon et al., 2023, in the case of RFID) claim to measure complex behaviors, including feeding and drinking, based solely on proximity data. Whether such inferences are always valid remains underexplored.

This study investigates whether the duration of chickens' presence near key resources in a mobile barn accurately reflects their actual usage. Specifically, we (1) delineate and highlight functional areas—where chickens can stay and interact with the resources—around 6 key facilities and (2) compare the manually collected durations of mere presence within these areas to actual resource use.

It is crucial to ensure that resource use proxies from modern tracking technologies are reliable. If these proxies do not accurately reflect actual resource use, data interpretation must be refined to maintain biological relevance.

## ANIMALS, MATERIALS AND METHODS

### Ethical Considerations

Our research complied with the German Animal Welfare Act, and necessary approvals were obtained from the competent authorities (authorization number AZ 7221.3-18196_23).

### Animals, Housing, and Husbandry

The study involved 21 chickens, a number chosen for practical reasons to enable efficient and feasible manual on-screen monitoring and focused observation. Furthermore, the low stocking density was intended to allow the chickens to perform their natural behaviors without hindrance from other chickens. The group included various lineages and sexes: 16 females and 1 male from the Vorwerk breed, all 68 wk old; 1 white Lohmann Selected Leghorn; and 3 Lohmann Brown chickens, all females and 45 wk old. The chickens had previously been housed together as a stable group for at least 12 wk. They were kept at a similar stocking density in a barn comparable to the one used in this study.

For this study, the chickens were housed in a ROWA 200 v4.0 mobile chicken barn at the “Friedrich harms” animal experimental station of the University of Rostock in Dummerstorf, Germany. The mobile barn was divided into 2 interconnected compartments: a 16-square-meter compartment with 6 Europa wooden nests, a 2.25-meter-long cylindrical metal perch, a 2-level L-shaped wooden perch, a 20 cm × 200 cm linear plastic trough feeder, and a drinker line with 3 bowls and 12 nipples. The other compartment, a 14-square-meter winter garden, offered a semi-open space with natural light, fresh air, and enrichment items like an alfalfa bale, mineral blocks, gravel, oyster shells, and a dust bath box. LED lighting provided 16 hours of light from 5:30 a.m. onwards.

We kept the chickens' management consistent with their prestudy conditions, providing the same commercial feed (PANTO LMK Legemehlkorn, Germany) and routine daily checks. No keel bone damage or severe feather pecking was observed during the study.

### Camera Configuration

Axis Communications cameras (M1135-E and M1135-E MK II), operating at 25 and 30 frames per second respectively, and equipped with 2-megapixel lenses (1920 × 1080), were used. The cameras were positioned on the ceiling of the chicken barn, with angles ranging from 29 to 90 degrees and a distance of up to 2.5 meters from the resources. The resources of interest included a drinker, nutritional enrichments in the winter garden, a feeder, a pophole, and metal and wooden perches.

### Video Recording and Segmentation

The recording period spanned 5 consecutive days (September 19-23, 2023). Video recordings for each resource were initially segmented into 10-min sequences, specifically capturing instances of animals interacting with resources throughout the day until the end of lighted hours. The number of manually segmented videos per resource were as follows: drinker (72), feeder (80), enrichment (80), wooden perch (72), metal perch (45), and pophole (45).

### Defining the Areas of Interest

**Figure 1:**
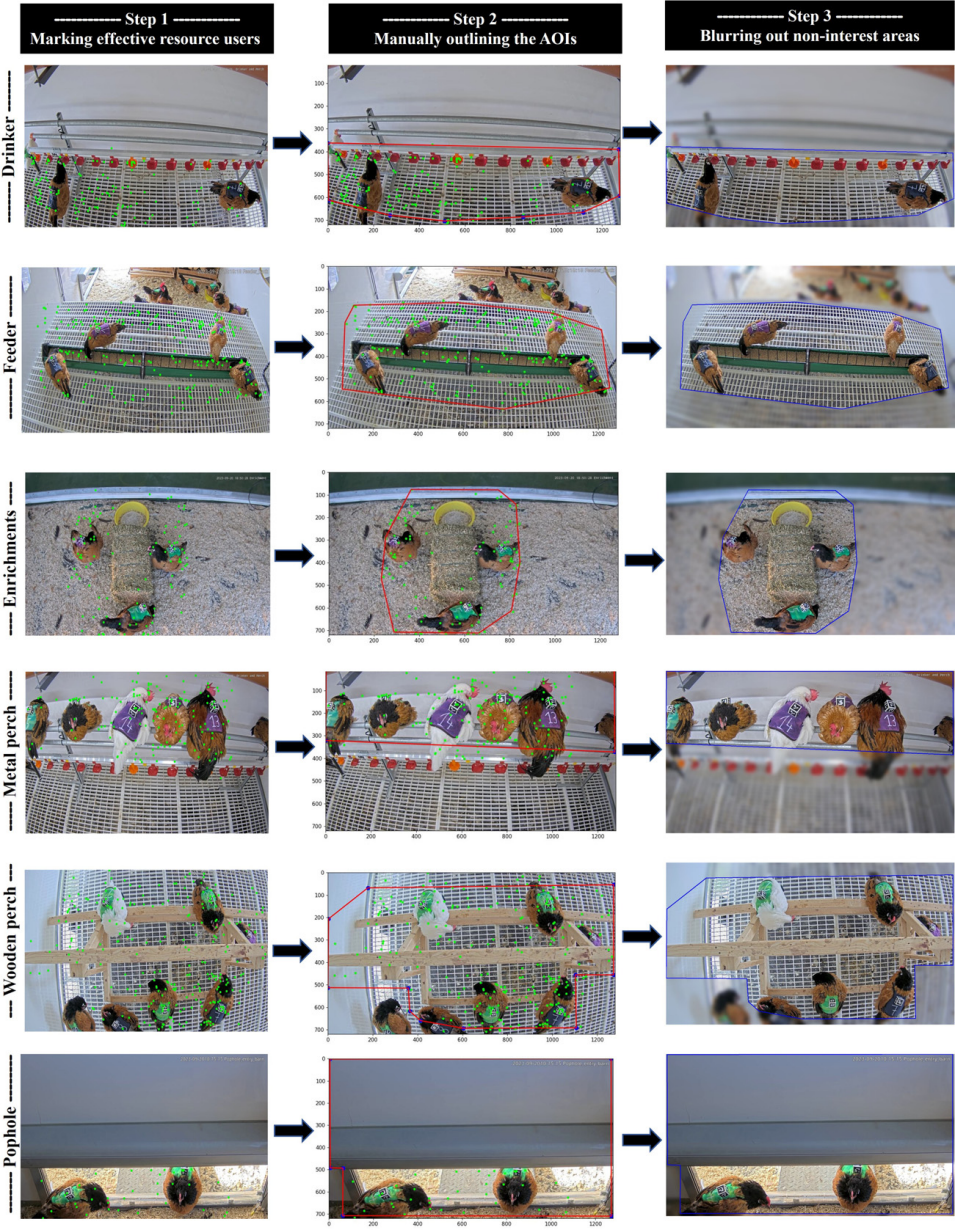
Images illustrating the 3 steps of defining areas of interest (AOIs) for each of the 6 key resources analyzed.

Defining the AOI, representing the functional areas of the resources where the chickens have the option to stay and interact with the resources, consisted of applying overlays to the segmented video sequences using Python's OpenCV library (v4.8.0). The process involved 3 steps (Figure 1).

First, effective resource users were identified. This step involved extracting video frames showing chickens actively using the resources and manually marking and collecting coordinates of key points on these chickens. Key points included the middle of the back and extremities, such as the tail tips, flanks, and heads. The points were marked for the following number of effective resource users: drinker (41), feeder (157), enrichment (40), wooden perch (129), metal perch (89), and pophole (21). The coordinates were then superimposed onto a reference image, forming a cloud of points that represented the main activity area of the chickens interacting with the resources.

Next, the area of interest was outlined by drawing a polygon that encompassed most of the marked points. It is important to note that the size of the AOIs varied between resources due to the inherent specifics of the targeted behaviors, as well as the position and placement of chickens effectively using the resource in the marked frames.

Finally, noninterest areas were blurred out, and the overlay was placed on the videos. This process ensured that only the chickens within the AOI were distinctly visible for annotations of proximity and effective resource use.

### Manual Annotations

Manual annotations were performed using the Behavioral Observation Research Interactive Software (BORIS v8.21.5) to accurately code the start and end timestamps for each individual's effective resource use and presence within the functional area around the resources (AOI). Since the chickens were equipped with colored vests (Omlet, UK), each bearing unique handwritten IDs visible on screen, it was possible to differentiate between them within each 10-min video sequence.

For annotation, it was essential to follow each individual throughout each sequence, focusing on the ID. This approach necessitated re-watching and annotating the same video sequence for as many different individuals as appeared during the 10-min intervals. Although differentiation of individuals within a 10-min sequence was possible, continuous tracking of individuals between different 10-min sequences was not feasible because the vests were redistributed each new day. Two ethograms were utilized: one to code mere presence, and another to code effective resource use.

*Ethogram for mere presence.* Mere presence was defined as the simple presence of a chicken within the AOI, irrespective of its activities and purposes for being there. Annotation began as soon as at least half of the chicken's body was within the AOI or if both feet were located in the defined AOI.

*Ethogram for effective resource use.* Effective resource use was recorded when chickens interacted with the resource based on specific criteria for each type of resource:

• Drinking: observed standing in front of the drinking line, the chicken might have dipped its beak into a cup, come into contact with a nipple drinker, or lifted its head to drink water by gravity. If after such active engagement with the water resource, the chicken paused—indicated by immobility without any other activity—and then resumed drinking, the same drinker use event continued. However, if during this time, the chicken engaged in other behaviors such as preening, social interaction, or environmental exploration, or if it left the defined AOI, the previous drinker use event was considered to have ended from the moment the animal ceased interacting with the drinker.
• Feeding: observed with its head tilted downwards, the chicken may actively peck at the feed either through a series of coordinated movements or with isolated pecks, or simply focus on it while in this position. Similarly to the drinking behavior described earlier, brief pauses can also be part of the same feeding event, and the criteria for initiating an effective feeder use event follow the same principles as described for drinking behavior.
• Perching: effective use of perches was recorded when chickens were either sitting or standing on the metal or wooden perch.
• Passing through the Pophole: effective use of the pophole was defined as chickens passing through the access hatch, transitioning from the main compartment to the covered winter garden, or in the opposite direction.
• Interactions with Nutritional Enrichments: effective use was recorded when chickens were observed pecking at enrichment items such as pecking stones, oyster shells, or an alfalfa bale, or showing interest in the fragments of these items on the ground nearby. Concerning the criteria for initiating an effective enrichment use event, the principles established for drinking behavior also apply here.

*Human annotators.* Two previously trained annotators coded the video sequences. Postannotation inter-rater reliabilities were calculated for the 2 utilized ethograms, with Cohen's kappa coefficients at 0.94 for resource use and 0.95 for mere presence within AOIs.

### Data and Statistical Analyses

*Variables and data handling.* For each of the 6 key resources, 35 sequences were randomly selected from the total segmented sequences obtained, ensuring representativeness and complete coverage of the 5-day recording period conducted on all 21 chickens. We made sure to include sequences from various times of the day—early morning, midday, and evening. Each sequence was treated as a separate observation unit for subsequent statistical analyses.

Each sequence was broken down into individual seconds to determine, for each tracked individual, the number of time points where mere presence matched or mismatched effective resource use. This allowed for the calculation of usage-to-presence ratios and the investigation of correlations between these 2 measurements.

*Comparing presence and effective use.* Statistical analyses were performed using “scipy.stats” (v1.11.1) in Python (v3.10.9). Spearman's correlation analysis was employed to examine how the duration of presence in AOIs (visit event) relates to the aggregate duration of effective resource use during each visit event. Kruskal-Wallis and Dunn's tests were employed to examine significant differences in usage-to-presence ratios among resources.

## RESULTS AND DISCUSSION

### Examining Presence vs. Effective Use Across Various Resources

**Figure 2:**
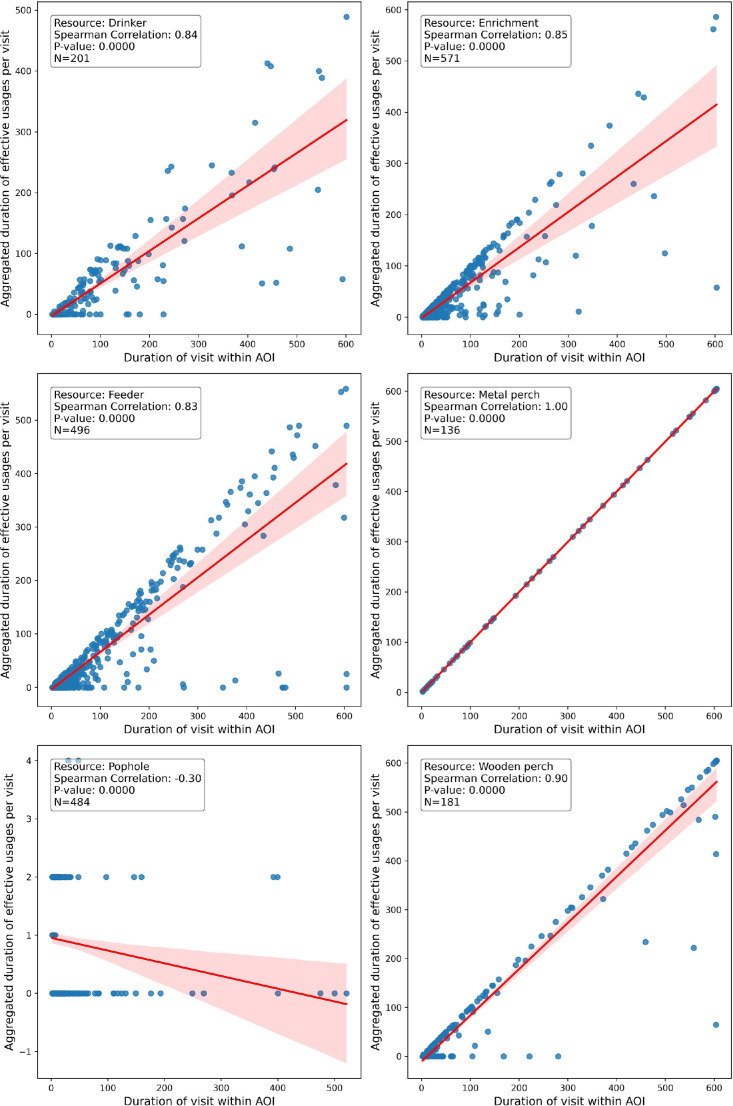
Correlation between the duration of visits in functional areas (i.e., Areas of Interest; AOIs) around key resources in the mobile barn and the aggregated duration of effective use within those visits. Each blue point represents a variable pair. The red line depicts the trend via a fitted regression line, while the pink band marks the 95% confidence interval.

This study investigated the relationship between chickens' proximity to key resources in a mobile poultry barn and their actual usage, assessing whether proximity serves as a reliable indicator of resource use. We found particularly strong Spearman's correlations (ranging from 0.83 to 1, *P* < 0.001) between chicken presence and resource use for most resources analyzed, except for the pophole, which displayed a much lower correlation (Rho = −0.30, *P* = 0.01; Figure 2).

Further analysis showed that the ratio of effective use to mere presence varied significantly across resources (H-test = 162.23, N = 210; *P* < 0.01), with metal and wooden perches resulting in the highest usage-to-presence ratios, exceeding 87%. Enrichments and feeder exhibited intermediate values around 66%, while drinker, statistically similar to the latter, had a usage-to-presence ratio of 50%. The pophole distinctly showed the lowest ratio, about 10%.

These findings underscore that proximity to a resource does not always translate into effective use in a poultry barn. This discrepancy highlights the need to interpret proximity data to infer effective resource use with caution, particularly for certain types of resources. While presence data can provide a reliable estimate of perch use, inferring feeding and drinking behaviors solely based on proximity data requires more caution, as it can lead to a significant overestimation of the actual use of feeders, nutritional enrichments, and drinkers. This caution is even more critical for popholes, where the ratios of usage relative to presence were notably low, indicating that proximity data alone—especially from one side of the pophole—is insufficient for accurate assessment.

The strong relationship between presence and usage of perches can likely be attributed to the elevated nature of the perches and the more focused coverage of their AOIs, which concentrate on the perch line with less overlap with other noninterest areas. The usage-to-presence ratio is even stronger if the perch is a single, high, isolated line, ensuring that only perching animals are captured, as the AOI can exclude all chickens underneath. This implies that technologies capturing presence, such as RFID, would be reliable if they are properly configured to ensure the reading range exclusively covers the perch line in height, as done by Wang et al. (2019).

The less strong relationship between proximity and usage for drinker, feeder, and nutritional enrichments can be explained by the fact that chickens approaching these resources often interrupt usage episodes with other activities, or engage exclusively in other activities not directly related to the resource itself, such as remaining still, exploring the environment, resting, crossing the area, preening, and social interactions. This suggests caution when using presence data to infer effective use of those resources. Should presence-tracking technology be used, it would need to be complemented with additional methods to reliably provide information on usage. These complementary measures could include water level sensors and flow meters for water intake, as well as environmental modifications to better track resource use (Tang et al., 2021). Additionally, machine learning algorithms, such as You Only Look Once (**YOLO**) demonstrated by Nasiri et al. (2023), could significantly enhance the accuracy of feeding behavior measurements.

The weak relationship between presence and usage of the pophole is likely due to chickens quickly passing through it for usage, while their presence generally consists of spending long periods sitting in the passageway, as observed by Richards et al. (2011) using RFID. This finding shows that focusing exclusively on presence data, particularly from one side of the pophole, may not be reliable. To reliably capture pophole usage, a more thorough approach, such as monitoring both sides of the passageway, is needed. With RFID, this would necessitate antennas positioned on both sides of the pophole—one to log entries and the other to register exits—prior to confirming their passage, whether directly (Gebhardt-Henrich et al., 2014) or through a modified pophole transformed into a tunnel equipped with RFID antennas at both the entrance and exit (Thurner et al., 2010). Alternatively, rather than solely monitoring pophole usage through tracking entries and exits, technologies could be employed to directly monitor animal presence in outdoor areas (e.g., winter garden).

### Limitations and Future Research

This study has notable limitations that need to be addressed for broader applicability. We focused on a single small flock housed in a mobile barn, limiting the generalizability of the findings. Future research is needed to expand the scope to include larger sample sizes and multiple flocks. Additionally, it is essential to consider conditions representative of commercial husbandry systems, where higher density and limited space per animal prevail. In such systems, the relationship between mere presence and effective resource use may be even weaker due to mobility limitations and competition for space, leading birds to occupy any available areas regardless of resource availability (Leone and Estévez, 2008).

Moreover, there was no exploration of how bird characteristics affected usage-to-presence ratios. The flock was idiosyncratic, with mixed breeds, ages, and sexes, which could have influenced individual and group behaviors. Understanding the dynamics within the flock, including social interactions, health status, and behavioral traits like aggression or fear levels, could provide deeper insights. Future studies should consider these factors to better interpret the relationship between presence and resource use.

Additionally, the way functional areas of interest are defined may impact the usage-to-presence ratios. In this study, functional areas were defined by marking areas where effective users were observed. However, other methods can also be considered. Future studies should statistically examine how different approaches, including variations in the size or shape of these areas, might affect the usage-to-presence ratios.

Lastly, while this research note discussed points particularly relevant for RFID and image-based tracking systems, similar investigations should be considered using other tracking technologies like accelerometers and thermal imaging.

Overall, this research note examined the relationship between the presence of chickens near key resources in a mobile poultry barn and their actual use. We showed that while presence detection can provide information about resource use, relying solely on presence data can overestimate actual resource use, particularly for certain resources such as feeders, enrichments, drinkers, and popholes. Our study highlights the need to systematically evaluate data collected from modern tracking technologies to ensure their validity and reliability in studying animal behavior.

## DISCLOSURES

The authors declare that they have no known competing financial interests or personal relationships that could have appeared to influence the work reported in this paper.
